# Using a mobile unit to deliver capsule sponge testing for Barrett’s oesophagus in the community in England through GP invitation and self-referral: an implementation study

**DOI:** 10.1136/bmjgast-2026-002496

**Published:** 2026-09-22

**Authors:** Rebecca Landy, Jo Waller, Irene Debiram-Beecham, Abigail Kerridge, James Booth, Peter Holloway, James Morrow, Fiona M Walter, Mimi McCord, Peter D Sasieni, Rebecca C Fitzgerald, Oliver Stovin

**Affiliations:** 1 Wolfson Institute of Population Health, Barts and The London School of Medicine and Dentistry, Queen Mary University of London, London, UK; 2 Surveillance, Prevention, & Health Services Research, American Cancer Society, Atlanta, Georgia, USA; 3 Cancer Prevention Group, School of Cancer and Pharmaceutical Sciences, King’s College London, London, UK; 4 Department of Oncology, Early Cancer Institute, University of Cambridge, Cambridge, UK; 5 Cambridge University Hospitals NHS Foundation Trust, Cambridge, UK; 6 Melbourne House Surgery, Parkside Medical Centre, Chelmsford, UK; 7 East of England Cancer Alliance, Cambridge, UK; 8 Granta Medical Practices, Cambridge, UK; 9 The Primary Care Unit, Department of Public Health and Primary Care, University of Cambridge, Cambridge, UK; 10 Heartburn Cancer UK, Basingstoke, UK; 11 Macmillan GP, Cambridge and Peterborough CCG, Cambridge, UK

**Keywords:** BARRETT'S OESOPHAGUS, SCREENING, OESOPHAGEAL CANCER

## Abstract

**Objective:**

Offering individuals with gastro-oesophageal reflux a non-endoscopic capsule sponge test increased detection of Barrett’s oesophagus (BO), a precursor to oesophageal adenocarcinoma. However, only 24% of individuals identified through general practitioner (GP) records participated. We therefore performed an implementation study to assess the feasibility of screening on a mobile unit and screening uptake among GP-invited and self-referred individuals.

**Methods:**

In this implementation study, 3293 individuals aged ≥50 with a repeat proton pump inhibitor prescription were invited to participate by their GPs across three GP practices in the East of England between June 2021 and January 2023. Additionally, we advertised the study and allowed self-referrals. Eligibility was assessed based on age and reflux history. Eligible individuals were invited to an appointment. Acceptability was assessed after the appointment. Individuals with a positive capsule sponge test were referred for endoscopy.

**Results:**

Half of the GP-invited individuals responded, of whom 59.6% (n=952/1598) were interested. Among interested individuals who could be contacted, 66.3% (n=574/866) were eligible, and 532 participated. Of these, 502 (94%) successfully swallowed a capsule sponge, representing 15% of those invited and 87.5% of eligible individuals. Among 468 individuals who self-referred and could be contacted, 48.5% (n=227) were eligible, 217 participated, and 208 (96%) successfully swallowed a capsule sponge (39.6% of self-referred, 91.6% of eligible individuals). 9.4% (n=47/502) of GP-invited and 12.0% (n=25/208) of self-referred individuals were referred for endoscopy, with 4.2% (n=21/502) and 3.4% (n=7/208), respectively, diagnosed with BO (positive predictive value of endoscopy referral=42.4%), including three with dysplasia. Over 99% of participants said the capsule sponge test was acceptable.

**Conclusion:**

Screening on a mobile unit is feasible. Identifying individuals at increased risk of BO from GP records is feasible, and encouraging self-referrals increases uptake. BO prevalence was similar among eligible participants who self-referred and those identified through GP records.

**Trial registration number:**

ISRCTN91655550.

**WHAT IS ALREADY KNOWN ON THIS TOPIC**

- It is not feasible to offer endoscopic screening for Barrett’s oesophagus (BO) to all patients with gastro-oesophageal reflux disease (GORD) in the UK; however, capsule sponge tests are minimally invasive and may offer an opportunity to screen larger numbers of people. It is unclear whether people with GORD would participate in or self-refer for this type of screening. Since performing capsule sponge screening requires special training, mobile units are a practical way to screen people in multiple geographical areas. This study invited eligible individuals through general practitioner records and encouraged self-referral to assess uptake.

**WHAT THIS STUDY ADDS**

- Two-thirds of invited individuals who responded were eligible, and 88% of those successfully swallowed a capsule sponge. Additionally, people were willing to self-refer, and the proportion diagnosed with BO was similar in both groups (4.2% of invited, 3.4% of self-referrers).

**HOW THIS STUDY MIGHT AFFECT RESEARCH, PRACTICE OR POLICY**

- Screening on a mobile unit is feasible and will be used in the BEST4 (Barrett’s oESophagus Trial 4) Screening Trial of capsule sponge screening, which aims to determine whether capsule sponge screening reduces oesophageal cancer mortality.

## Introduction

 In the UK, approximately 9300 oesophageal cancer cases are diagnosed each year, mostly adenocarcinomas.^1 2^ Gastro-oesophageal reflux disease (GORD) is a risk factor for oesophageal adenocarcinoma (OAC),^3 4^ with the precursor Barrett’s oesophagus (BO) occurring in 3–6% of individuals with GORD.^5^ Regular endoscopic surveillance of patients with BO is recommended, with the aim of (1) identifying and treating dysplastic BO, preventing progression to cancer; and (2) reducing mortality through earlier diagnosis of cancers that are not prevented.^6–8^

Given the high prevalence of reflux symptoms, screening all patients with GORD for BO using endoscopy is not feasible.^9^ The current British Society of Gastroenterology guidelines^10^ therefore only recommend endoscopy for individuals with GORD and three or more of the following: age ≥50, white race, male sex, and body mass index (BMI) ≥30 kg/m^2^. However, they do not recommend a proactive effort to identify individuals who meet these criteria. The National Institute for Health and Care Excellence guidelines on the management of individuals with GORD suggest discussing the person’s preferences and individual risk factors before recommending endoscopy.^11^

Capsule sponge tests are minimally invasive, non-endoscopic cell collection devices, coupled with a laboratory test for the trefoil factor 3 biomarker, developed to detect intestinal metaplasia, a hallmark of BO.^5 12^ The 10-minute office-based procedure is safe,^13–15^ acceptable to patients,^5 15–17^ and has good diagnostic accuracy.^5 13 18^ Individuals testing positive are referred for a diagnostic endoscopy. In a randomised controlled trial, offering capsule sponge screening to individuals with GORD detected 10 times more BO than usual care.^5^ However, only 24% of general practitioner (GP)-invited individuals identified through prescribing records accepted the offer of a capsule sponge test.^5^ The optimal way to identify individuals who may benefit and encourage uptake is not known. Furthermore, it is not known whether individuals who are not invited would self-refer for a capsule sponge test, what proportion of self-referrers would be eligible, or how the BO positivity rate among these individuals would compare with that among those invited by their GPs.

In this study, we assessed the feasibility of performing capsule sponge screening in mobile units, uptake among individuals invited by their GPs, and participation among individuals who self-referred. We report the characteristics of DELTA (integrated Diagnostic solution for Early detection of Oesophageal cAncer) participants who attended the mobile units, stratified by their recruitment method, as well as the proportions who had BO and the logistics of screening in a mobile unit.

## Methods

### Study design

Two changes were made from the original study design outlined in the study registration. Project DELTA aimed to assess the feasibility of implementing capsule sponge screening in hospitals, primary care and community settings. However, due to the COVID-19 pandemic, this was changed to assess the feasibility of screening in a mobile unit, after mobile units were successfully used for the lung cancer screening pilot in England.^19^ Additionally, at the request of patient advocates and Heartburn Cancer UK, individuals were allowed to self-refer for this study.

For this implementation study, individuals aged ≥50 with a repeat proton pump inhibitor (PPI) prescription were identified via electronic health records from three general practices in the East of England between June 2021 and January 2023. Exclusion criteria are reported in box 1. The search codes for identifying individuals were expanded during the study to allow for differences in how things were coded between sites. Participants identified through medical records received a letter from their GPs inviting them to return a reply slip if interested in a capsule sponge test at a local mobile unit; those not interested could indicate their reason using a tick box or free text.

Box 1 **Exclusion criteria**

- Recorded upper gastrointestinal endoscopy for any reason within the past 5 years.*
- Barrett’s oesophagus.
- Diagnosis of current or previous oropharyngeal, oesophageal or gastro-oesophageal tumour.
- Under current hospital care.
- Referred for hospital care for any reason.
- Met the 2-week wait guidance for oesophageal cancer.^30^
- Proton pump inhibitor (PPI) was prescribed for protection alongside non-steroidal anti-inflammatory drugs or steroids.
- Bleeding tendency.
- Oesophageal varices or cirrhosis of the liver.
- Previous surgical intervention to the oesophagus.
- Difficulty swallowing.
- Unable to temporarily discontinue antithrombotic medication prior to procedure (in line with the manufacturer’s guidance).
- Lacks the capacity to provide informed consent.

*This was briefly 2 years but was updated during recruitment at the first site.

The second recruitment route was via self-referral in the same geographical areas as the three GP practices mentioned above. The study was advertised on local community websites and Facebook pages, and adverts were placed in pharmacies and larger shops in the town centres. Interested people aged ≥40 taking over-the-counter medication for reflux who were not having their symptoms investigated were asked to contact the study team. Apart from age, the exclusion criteria were the same as for those invited by their GPs. The lower age limit was at the request of Heartburn Cancer UK, who provided the mobile unit, due to the increasing numbers of individuals diagnosed below age 50, despite the low absolute risk in this population. It is possible that some individuals were exposed to both recruitment methods.

Those expressing interest via either route were assessed for eligibility in a phone call.

Written informed consent was obtained from all participants prior to the capsule sponge procedure.

### Study sites

Appointments took place at three sites in the East of England: Shelford (on the outskirts of Cambridge); Chelmsford, a city in Essex; and Eye, a market town in Suffolk. The Shelford general practice was in the least deprived decile^20^ in an urban area (2011 Census Rural-Urban Classification C1: urban city and town); the Chelmsford general practice was in the third most deprived decile in an urban area (2011 Census Rural-Urban Classification C1: urban city and town); and the Suffolk general practice was based in the sixth most deprived decile in a rural area (2011 Census Rural-Urban Classification D1: rural town and fringe). The mobile unit was active in Shelford between June 2021 and December 2021; in Chelmsford between February 2022 and June 2022; and in Eye between September 2022 and January 2023. The sites were selected to provide a range of deprivation levels and rurality. For additional details on the sites, see the online supplemental methods. A schematic of the mobile unit is provided in online supplemental figure 1 and a photograph in online supplemental figure 2.

### Study procedures

Participant eligibility was reconfirmed on arrival. The procedure, risks, and benefits were described; the participant was shown the capsule sponge device (Cytosponge from Medtronic Inc) and given the opportunity to ask questions prior to providing consent. Age, sex, ethnicity, smoking status, height, weight, and duration of antireflux medication were recorded (unless recorded during the screening call). Deprivation was measured using the Index of Multiple Deprivation (IMD) based on the participant’s home address. Participants completed a questionnaire after the procedure, including two questions on acceptability (see the online supplemental methods). They were offered a sweet after their procedure to help with any residual discomfort in the throat. After the samples were analysed, individuals whose capsule sponge did not collect any columnar cells (suggesting an incomplete swallow that did not reach the gastric cardia) were invited for a repeat test. Those with a positive result were invited for endoscopy at the nearest hospital assigned to the study. Endoscopy follow-up was available until April 2023.

The mobile unit was staffed by two individuals, at least one of whom was a nurse; the other was a nurse, project manager or administrator. Although the mobile unit was not wheelchair accessible, if a patient required this, they were seen in a clinic room nearby, for example, in the first aid room at the sports centre in Chelmsford. Up to 15 appointments could be scheduled per day, though 8–12 was standard. Overall, individuals did not show up for <1% of the scheduled appointments due to telephone reminders. Over 80% of appointments lasted between 16 and 30 min, with 9% lasting <15 min. Eight of the 18 (44%) people whose appointment took more than 35 min required more than one attempt to swallow the capsule sponge or were not able to swallow it.

### Statistical methodology

The primary study outcomes were the proportion of individuals contacted by their GPs who participated in the study, the number of participants recruited via self-referral, and the proportion of screened patients who were diagnosed with BO. Descriptive analyses were performed to test for differences in characteristics by referral method (GP-invited vs self-referral). T-tests were used to test for differences in distributions between continuous variables (age), Mann-Whitney tests for ordinal variables (medication duration), a test for differences in proportions (with CIs calculated using the Wilson score method^21^) for binary variables (sex, ethnicity (white British vs other), and deprivation (quintile (Q1–Q3 vs Q4–Q5)), and χ^2^ tests for categorical variables (grouped BMI, smoking status). Univariate logistic regression was used to determine whether any characteristics were associated with the odds of a BO diagnosis. Individuals with missing data were excluded from statistical tests and logistic regression modelling. The Standards for Reporting Implementation Studies (StaRI) reporting guideline was followed (online supplemental file 2).^22 23^

## Results

### Study population

**Figure 1:**
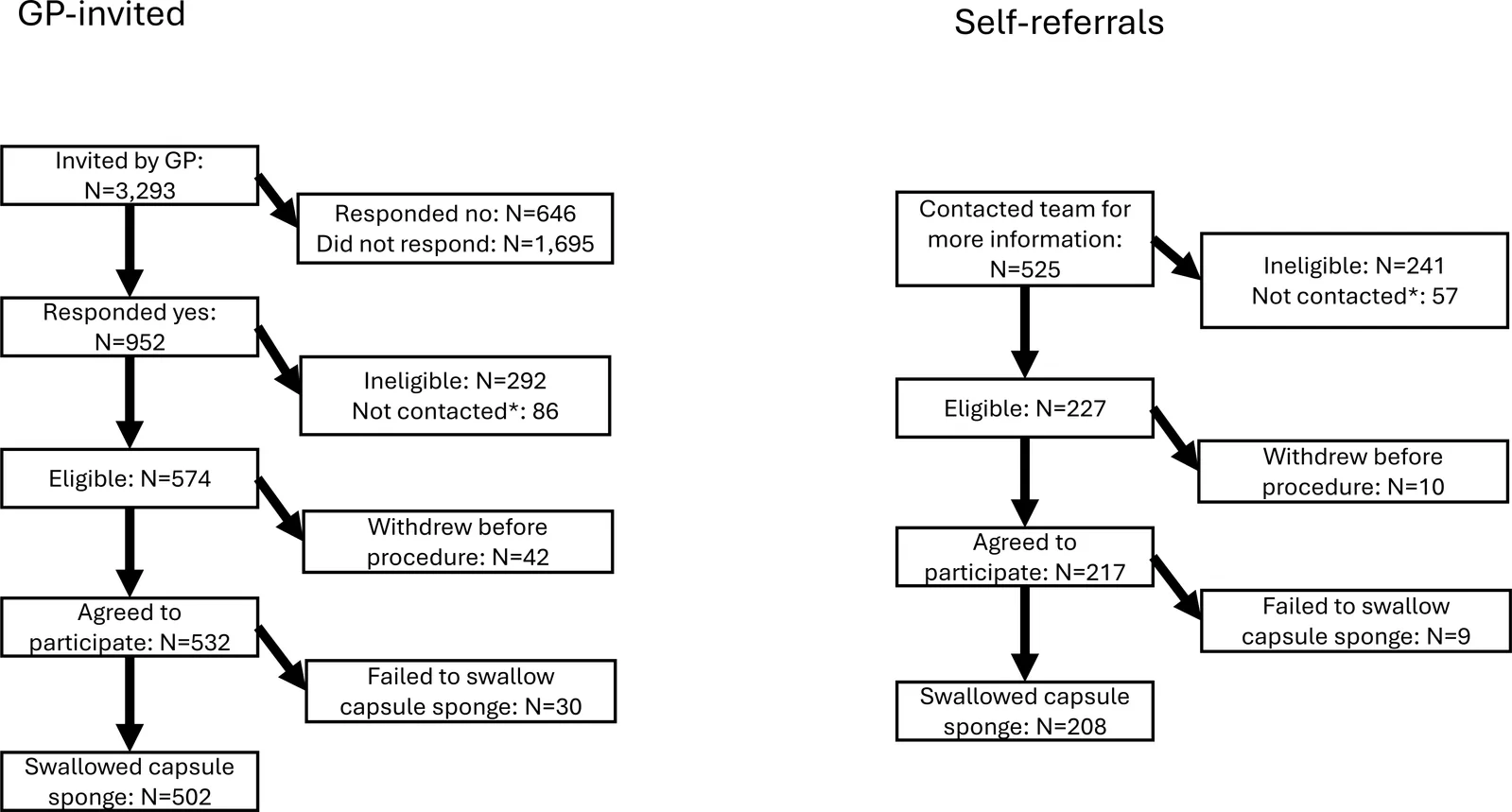
Flow of individuals through the study, stratified by whether they were invited by their general practitioners (GPs) or self-referred. *Could not be contacted or were contacted once the mobile unit was no longer local, or they changed their mind after initially expressing interest.

Overall, 3293 individuals were invited by their GPs (GP-invited; figure 1, online supplemental table 1). 1598 individuals (48.5%, range across sites 42.2–52.5%) returned a slip, of whom 952 (59.6%, range 51.4–66.1%) expressed interest in the study. Almost one-tenth of individuals who expressed interest could not be contacted while the mobile unit was still local or changed their mind (86/952, 9.0%). Of those who were interested and could be contacted, 574 (66.3%, range 58.6–78.1%) were eligible, and 532 (92.7%, range 84.2–100.0%) agreed to participate. Of these, 502 (94.4%, range 93.6–95.7%) successfully swallowed a capsule sponge, corresponding to 15.2% (range 10.6–20.9%) of individuals invited to participate and 87.5% (range 80.5–93.6%) of interested individuals who were eligible.

Additionally, 525 individuals who had not been invited by their GPs contacted the study team for additional information (self-referrals; figure 1): 191 (36.4%) in Shelford, 86 (16.4%) in Suffolk and 248 (47.2%) in Chelmsford. A similar proportion to those referred by their GPs could not be contacted while the mobile unit was still in the local area or changed their mind (57/525, 10.9%). Of those who could be contacted in time, 48.5% were eligible (n=227), with similar proportions at each site (Shelford: 49.4%; Suffolk: 47.4%; Chelmsford: 48.2%). 217/227 agreed to participate, and 208 (95.9%) successfully swallowed a capsule sponge, corresponding to 39.6% (range 38.4–40.3%) of those who contacted the study team. In total, 710 individuals successfully swallowed a capsule sponge, of whom 29.3% self-referred. In total, 665/710 (92.3%) successfully swallowed a capsule sponge on their first attempt. Individual-level data on invited individuals who did not respond or did not want to participate were not available. Reasons for non-participation and ineligibility are discussed below.

### Comparing characteristics of GP-invited versus self-referred individuals

Among individuals who attempted to swallow a capsule sponge, self-referrals were younger than GP-invited (p<0.001); this partly reflects the younger age eligibility criteria for self-referrals (table 1). A non-statistically significantly higher proportion of self-referrals were women (self-referrals 57% vs GP-invited 49%, p=0.071). Self-referrals had a shorter history of antireflux medication use compared with GP-invited individuals (p<0.001). Additionally, a higher proportion of self-referrals with known ethnicity were not white British (8.8% vs 3.0%, p<0.001). There were no significant differences in the smoking status (p=0.559), IMD (p=0.150 for Q1–Q3 vs Q4–Q5) or BMI (p=0.794) of self-referrals compared with GP-invited individuals.

**Table 1:** Characteristics of individuals who attempted to swallow a capsule sponge device, by invitation status and study site

	Total n (%)	GP-invited				Self-referrals			
		Chelmsford n (%)	Shelford n (%)	Suffolk n (%)	Total n (%)	Chelmsford n (%)	Shelford n (%)	Suffolk n (%)	Total n (%)
Total	749 (100)	140 (100)	186 (100)	206 (100)	532 (100)	103 (100)	79 (100)	35 (100)	217 (100)
Age									
<50	39 (5.2)	0 (0.0)	0 (0.0)	0 (0.0)	0 (0.0)	22 (21.4)	9 (11.4)	8 (22.9)	39 (18.0)
50–59	166 (22.2)	23 (16.4)	56 (30.1)	13 (6.3)	92 (17.3)	40 (38.8)	29 (36.7)	5 (14.3)	74 (34.1)
60–69	259 (34.6)	49 (35.0)	67 (36.0)	80 (38.8)	196 (36.8)	22 (21.4)	28 (35.4)	13 (37.1)	63 (29.0)
70–80	273 (36.4)	62 (44.3)	62 (33.3)	110 (53.4)	234 (44.0)	19 (18.4)	12 (15.2)	8 (22.9)	39 (18.0)
>80	1 (0.1)	0 (0.0)	0 (0.0)	0 (0.0)	0 (0.0)	0 (0.0)	1 (1.3)	0 (0.0)	1 (0.5)
Missing	11 (1.5)	6 (4.3)	1 (0.5)	3 (1.5)	10 (1.9)	0 (0.0)	0 (0.0)	1 (2.9)	1 (0.5)
Gender									
Male	362 (48.3)	62 (44.3)	83 (44.6)	124 (60.2)	269 (50.6)	36 (35.0)	35 (44.3)	22 (62.9)	93 (42.9)
Female	385 (51.4)	78 (55.7)	103 (55.4)	81 (39.3)	262 (49.2)	67 (65.0)	43 (54.4)	13 (37.1)	123 (56.7)
Other/missing	2 (0.3)	0 (0.0)	0 (0.0)	1 (0.5)	1 (0.2)	0 (0.0)	1 (1.3)	0 (0.0)	1 (0.5)
Race									
White British	681 (90.9)	110 (78.6)	172 (92.5)	205 (99.5)	487 (91.5)	95 (92.2)	65 (82.3)	34 (97.1)	194 (89.4)
Asian	6 (0.8)	0 (0.0)	4 (2.2)	0 (0.0)	4 (0.8)	1 (1.0)	1 (1.3)	0 (0.0)	2 (0.9)
Other	30 (4.0)	5 (3.6)	6 (3.2)	0 (0.0)	11 (2.1)	6 (5.8)	12 (15.2)	1 (2.9)	19 (8.8)
Missing	32 (4.3)	25 (17.9)	4 (2.2)	1 (0.5)	30 (5.6)	1 (1.0)	1 (1.3)	0 (0.0)	2 (0.9)
Smoking status									
Current	32 (4.3)	6 (4.3)	7 (3.8)	7 (3.4)	20 (3.8)	3 (2.9)	6 (7.6)	3 (8.6)	12 (5.5)
Former	338 (45.1)	70 (50.0)	68 (36.6)	103 (50.0)	241 (45.3)	52 (50.5)	29 (36.7)	16 (45.7)	97 (44.7)
Never	360 (48.1)	59 (42.1)	107 (57.5)	91 (44.2)	257 (48.3)	46 (44.7)	43 (54.4)	14 (40.0)	103 (47.5)
Missing	19 (2.5)	5 (3.6)	4 (2.2)	5 (2.4)	14 (2.6)	2 (1.9)	1 (1.3)	2 (5.7)	5 (2.3)
BMI									
<25	199 (26.6)	38 (27.1)	65 (34.9)	44 (21.4)	147 (27.6)	23 (22.3)	26 (32.9)	3 (8.6)	52 (24.0)
25 to <30	329 (43.9)	49 (35.0)	76 (40.9)	102 (49.5)	227 (42.7)	49 (47.6)	35 (44.3)	18 (51.4)	102 (47.0)
30+	213 (28.4)	50 (35.7)	43 (23.1)	59 (28.6)	152 (28.6)	31 (30.1)	17 (21.5)	13 (37.1)	61 (28.1)
Unknown	8 (1.1)	3 (2.1)	2 (1.1)	1 (0.5)	6 (1.1)	0 (0.0)	1 (1.3)	1 (2.9)	2 (0.9)
Duration on antireflux medication									
No medication	7 (0.9)	2 (1.4)	4 (2.2)	0 (0.0)	6 (1.1)	0 (0.0)	1 (1.3)	0 (0.0)	1 (0.5)
<1 year	51 (6.8)	7 (5.0)	3 (1.6)	10 (4.9)	20 (3.8)	18 (17.5)	9 (11.4)	4 (11.4)	31 (14.3)
1 to <3 years	98 (13.1)	19 (13.6)	23 (12.4)	24 (11.7)	66 (12.4)	17 (16.5)	8 (10.1)	7 (20.0)	32 (14.7)
3 to <5 years	97 (13.0)	17 (12.1)	20 (10.8)	22 (10.7)	59 (11.1)	21 (20.4)	12 (15.2)	5 (14.3)	38 (17.5)
5+ years	454 (60.6)	88 (62.9)	124 (66.7)	143 (69.4)	355 (66.7)	42 (40.8)	42 (53.2)	15 (42.9)	99 (45.6)
Missing	42 (5.6)	7 (5.0)	12 (6.5)	7 (3.4)	26 (4.9)	5 (4.9)	7 (8.9)	4 (11.4)	16 (7.4)
IMD quintile									
1 (lowest)	6 (0.8)	4 (2.9)	0 (0.0)	0 (0.0)	4 (0.8)	0 (0.0)	2 (2.5)	0 (0.0)	2 (0.9)
2	40 (5.3)	26 (18.6)	0 (0.0)	1 (0.5)	27 (5.1)	10 (9.7)	1 (1.3)	2 (5.7)	13 (6.0)
3	216 (28.8)	10 (7.1)	16 (8.6)	134 (65.0)	160 (30.1)	25 (24.3)	12 (15.2)	19 (54.3)	56 (25.8)
4	201 (26.8)	27 (19.3)	44 (23.7)	60 (29.1)	131 (24.6)	33 (32.0)	26 (32.9)	11 (31.4)	70 (32.3)
5 (highest)	241 (32.3)	73 (52.1)	85 (45.7)	9 (4.4)	167 (31.4)	34 (33.0)	37 (46.8)	3 (8.6)	74 (34.1)
Missing	45 (6.0)	0 (0.0)	41 (22.0)	2 (1.0)	43 (8.1)	1 (1.0)	1 (1.3)	0 (0.0)	2 (0.9)

BMI, body mass index; GP, general practitioner; IMD, Index of Multiple Deprivation.

### Outcomes among individuals who swallowed a capsule sponge

Of the 710 individuals who successfully swallowed a capsule sponge, 561 (79.0%) had a negative test and were discharged, 76 (10.7%) had an insufficient or equivocal result and were invited for a repeat capsule sponge, and 72 (10.1%) had positive findings and were referred for endoscopy (table 2). A similar proportion of individuals were referred for endoscopy among GP-invited (9.4%) and self-referrals (12.0%, p=0.352). Additionally, 3/43 were referred for endoscopy following a repeat capsule sponge test, resulting in 10.6% overall being referred for endoscopy. Five people did not respond to their invitation for a repeat capsule sponge, 25 declined a repeat capsule sponge, 2 were unable to swallow the capsule sponge, and 1 attended endoscopy instead.

**Table 2:** Action resulting from capsule sponge device results, by invitation status and study site

			Discharged	Repeat capsule sponge device	Referred endoscopy	Clinic appointment
GP-invited	Chelmsford	n	102	14	15	0
		%	77.9	10.7	11.5	0.0
	Shelford	n	135	29	14	0
		%	75.8	16.3	7.9	0.0
	Suffolk	n	155	20	18	0
		%	80.3	10.4	9.3	0.0
	Total	n	392	63	47	0
		%	78.1	12.5	9.4	0.0
Self-referrals	Chelmsford	n	82	4	11	1
		%	83.7	4.1	11.2	1.0
	Shelford	n	57	7	13	0
		%	74.0	9.1	16.9	0.0
	Suffolk	n	30	2	1	0
		%	90.9	6.1	3.0	0.0
	Total	n	169	13	25	1
		%	81.3	6.3	12.0	0.5
Total		n	561	76	72	1
		%	79.0	10.7	10.1	0.1

GP, general practitioner.

Of the 75 individuals referred for endoscopy and one who attended endoscopy instead of a repeat capsule sponge, three declined endoscopy, and seven were lost to follow-up (three unknown whether they had an endoscopy, four underwent endoscopy but the results are unknown). Nine of the 10 individuals who declined endoscopy or were lost to follow-up were recruited via their GPs, corresponding to 17.6% (9/51) of GP-invited individuals and 4% (1/25) of self-referrals. 28/66 individuals who underwent an endoscopy following their initial or repeat positive capsule sponge and had a known endoscopy result were diagnosed with BO (positive predictive value (PPV) 42.4%; 21/28 were GP-invited), including three with dysplastic BO (4.5%; 2/3 were GP-invited; one high-grade dysplasia, one low-grade dysplasia, one indefinite for dysplasia), which were actionable results. These correspond to 4.2% and 3.4% of GP-invited and self-referrals, respectively, who are known to have swallowed a capsule sponge (21/502 and 7/208, respectively, p=0.766). Assuming that individuals who were capsule sponge positive but did not have known endoscopy outcomes had the same rate of BO as other capsule sponge-positive individuals, this would correspond to 5.1% of GP-invited and 3.5% of self-referrals having BO, including 0.5% in each group having dysplastic BO. The number of individuals who swallowed a capsule sponge per person diagnosed with BO was 24 for those invited by their GPs and 30 for individuals who self-referred. None of the variables considered (age, sex, smoking status, BMI, duration on antireflux medication and IMD quintile) were significantly associated with a diagnosis of BO (table 3).

**Table 3:** Associations between individual characteristics and BO diagnosis among individuals with an adequate capsule sponge result

	Total				GP-invited		Self-referrals	
	BO diagnosis n (Row %)	No BO diagnosis n (Row %)	OR (95% CI) for BO diagnosis	P value	BO diagnosis n (Row %)	No BO diagnosis n (Row %)	BO diagnosis n (Row %)	No BO diagnosis n (Row %)
Total	28 (3.9)	682 (96.1)			21 (4.2)	481 (95.8)	7 (3.4)	201 (96.6)
Age								
<50	2 (5.7)	33 (94.3)	2.36 (0.32 to 12.64)	0.72	0 (–)	0 (–)	2 (5.7)	33 (94.3)
50–59	4 (2.5)	156 (97.5)	1 (ref)		3 (3.4)	86 (96.6)	1 (1.4)	70 (98.6)
60–69	11 (4.4)	237 (95.6)	1.81 (0.61 to 6.62)		8 (4.3)	179 (95.7)	3 (4.9)	58 (95.1)
70+	11 (4.3)	245 (95.7)	1.75 (0.59 to 6.41)		10 (4.6)	206 (95.4)	1 (2.5)	39 (97.5)
Missing	0 (0.0)	11 (100.0)			0 (0.0)	10 (100.0)	0 (0.0)	1 (100.0)
Gender								
Male	15 (4.2)	342 (95.8)	1.14 (0.53 to 2.47)	0.73	13 (4.9)	253 (95.1)	2 (2.2)	89 (97.8)
Female	13 (3.7)	338 (96.3)	1 (ref)		8 (3.4)	227 (96.6)	5 (4.3)	111 (95.7)
Other/missing	0 (0.0)	2 (100.0)			0 (0.0)	1 (100.0)	0 (0.0)	1 (100.0)
Ethnicity								
White British	24 (3.7)	621 (96.3)			18 (3.9)	442 (96.1)	6 (3.2)	179 (96.8)
Asian	0 (0.0)	6 (100.0)	n/a		0 (0.0)	4 (100.0)	0 (0.0)	2 (100.0)
Other	0 (0.0)	30 (100.0)	n/a		0 (0.0)	11 (100.0)	0 (0.0)	19 (100.0)
Missing	4 (13.8)	25 (86.2)			3 (11.1)	24 (88.9)	1 (50.0)	1 (50.0)
Smoking status								
Current	1 (3.2)	30 (96.8)	1 (ref)	0.88	0 (0.0)	19 (100.0)	1 (8.3)	11 (91.7)
Former	12 (3.6)	318 (96.4)	1.14 (0.21 to 21.04)		10 (4.3)	223 (95.7)	2 (2.1)	95 (97.9)
Never	15 (4.3)	331 (95.7)	1.35 (0.26 to 24.93)		11 (4.4)	237 (95.6)	4 (4.1)	94 (95.9)
Missing	0 (0.0)	3 (100.0)			0 (0.0)	2 (100.0)	0 (0.0)	1 (100.0)
BMI (kg/m^2^)								
<25	3 (1.6)	180 (98.4)	1 (ref)	0.2	2 (1.5)	131 (98.5)	1 (2.0)	49 (98.0)
25–30	15 (4.8)	300 (95.2)	3.00 (0.97 to 13.08)		12 (5.5)	205 (94.5)	3 (3.1)	95 (96.9)
30+	10 (4.9)	196 (95.1)	3.06 (0.92 to 13.81)		7 (4.7)	141 (95.3)	3 (5.2)	55 (94.8)
Unknown	0 (0.0)	6 (100.0)			0 (0.0)	4 (100.0)	0 (0.0)	2 (100.0)
Duration on antireflux medication								
No medication	1 (14.3)	6 (85.7)	4.00 (0.17 to 48.55)	0.31	1 (16.7)	5 (83.3)	0 (0.0)	1 (100.0)
<1 year	2 (4.0)	48 (96.0)	1 (ref)		1 (5.3)	18 (94.7)	1 (3.2)	30 (96.8)
1–3 years	6 (6.6)	85 (93.4)	1.69 (0.37 to 11.87)		5 (8.2)	56 (91.8)	1 (3.2)	29 (96.7)
3–5 years	1 (1.1)	92 (98.9)	0.26 (0.01 to 2.79)		1 (1.8)	55 (98.2)	0 (0.0)	37 (100.0)
5+ years	17 (3.9)	421 (96.1)	0.97 (0.27 to 6.23)		13 (3.8)	330 (96.2)	4 (4.2)	91 (95.8)
Missing	1 (3.2)	30 (96.8)			0 (0.0)	17 (100.0)	1 (7.1)	13 (92.9)
IMD quintile								
1 (lowest)	0 (0.0)	6 (100.0)			0 (0.0)	4 (100.0)	0 (0.0)	2 (100.0)
2	4 (10.8)	33 (89.2)	4.46 (1.06 to 17.72)	0.16	3 (12.0)	22 (88.0)	1 (8.3)	11 (91.7)
3	7 (3.4)	197 (96.6)	1 (ref)		7 (4.6)	145 (95.4)	0 (0.0)	52 (100.0)
4	5 (2.6)	184 (97.4)	1.31 (0.41 to 4.49)		4 (3.3)	118 (96.7)	1 (1.5)	66 (98.5)
5 (highest)	10 (4.4)	219 (95.6)	1.68 (0.59 to 5.48)		5 (3.2)	151 (96.8)	5 (6.8)	68 (93.2)
Missing	2 (4.4)	43 (95.6)			2 (4.7)	41 (95.3)	0 (0.0)	2 (100.0)

BMI, body mass index; BO, Barrett’s oesophagus; GP, general practitioner; IMD, Index of Multiple Deprivation.

### Acceptability

The capsule sponge device was very acceptable to participants, with only 4/713 individuals who attempted to swallow a capsule sponge device (0.6%) saying they found it unacceptable or completely unacceptable, three of whom were unable to swallow the capsule sponge. The full distribution of results is provided in the online supplemental results. 96.8% (692/715) of participants said they would definitely or probably be willing to have another capsule sponge procedure, with 67.1% (n=480/715) saying ‘definitely’. Only 1.5% of participants (n=11/715) said no.

### Reasons for non-participation

In total, 646 people (19.6%; range 17.8–20.5% across sites) invited by their GPs returned a slip confirming they were not interested in participating. The most endorsed reasons were not thinking they could swallow the capsule (208/646; 32.2%) and having other more important medical issues (195/646; 30.2%). Some of the less commonly endorsed reasons for not wanting to participate included preferring to have an endoscopy if symptoms needed investigating (68/646; 10.5%), preferring not to know about problems with their oesophagus (49/646; 7.6%), concern about COVID-19 (36/646; 5.6%) and being too busy to make an appointment (29/646; 4.5%).

Almost a quarter (158/646; 24.5%) of those who returned a slip believed themselves to be ineligible for the study for one or more reasons, including not having (or no longer having) reflux symptoms (102/646; 15.8%) and taking PPI medication for reasons other than reflux, for example, to control the side effects of another medication (55/646; 8.5%). Perceived ineligibility among those giving a reason for non-participation varied by site and decreased as the study progressed: 31.1% (47/151) in Shelford, 24.4% (192/254) in Chelmsford, and 20.3% (192/241) in Suffolk. This reflects some success from altering the search criteria during the Shelford study to reduce the number of inappropriate individuals identified, for example, by expanding the codes used to identify individuals who had received an endoscopy. Additionally, 50 individuals invited by their GPs who initially expressed interest in the study changed their mind after being contacted by the study team, as did 24 individuals who self-referred.

### Reasons for ineligibility

We were able to classify reasons for ineligibility for 436/533 (82%) individuals: 279/292 (96%) GP-invited and 157/241 (65%) self-referrals (online supplemental table 2). The main reasons GP-invited individuals were ineligible for the study were having had an endoscopy within the past 5 years (34%), taking a PPI as a coprescription rather than due to reflux symptoms (18%) and having a comorbidity (17%), whereas self-referrals were more likely to be ineligible for not having taken a PPI for at least 6 months due to GORD (43%), having had an endoscopy within the past 5 years (34%) and having a comorbidity (12%). The search criteria to identify individuals from GPs were altered during the Shelford study with the goal of lowering the proportion of people who were ineligible. The proportion of ineligible individuals who were not on PPIs/had insufficient reflux symptoms or who had PPIs coprescribed was lower in the other two sites, though there were increases in the proportion who had a recent endoscopy or who were on contraindicated medications (online supplemental table 2). Reasons for ineligibility likely reflect the characteristics of the local population and are expected to vary by GP practice. Additionally, the lack of standardisation of clinical codes used both within and between GP practices will likely contribute to differences in reasons for ineligibility.

## Discussion

### Summary

DELTA was the first study using a mobile unit to deliver capsule sponge testing to people at risk of BO, and it showed that it is feasible and acceptable. In addition, encouraging individuals to self-refer in response to local advertising was successful, and participants who self-referred had similar rates of BO as those invited by a GP.

This study confirmed findings from the BEST3 (Barrett’s oESophagus Trial 3) study, which demonstrated that it was feasible to use GP records to identify individuals for BO screening, though only half those identified responded to the invitation, and fewer than one in six of those invited swallowed a capsule sponge device. This was slightly lower than in BEST3 (15% vs 24%), driven by the lower proportion expressing interest in the study in response to the initial invitation (29% vs 39%). This study took place during the COVID-19 pandemic, which likely discouraged people from participating. Almost one-third of individuals who gave a reason for their non-participation did not think they could swallow the capsule. The majority of individuals would not previously have been familiar with capsule sponge testing, and although the procedure was described and videos are available online, a direct link to a video showing the procedure has been included in the BEST4 Screening Trial, which we hope will reduce the proportion of people who think they would not be able to complete the procedure, as may telling them the proportion of people who swallow it successfully. We note that capsule sponge testing is not yet standard of care for people with reflux symptoms in England; if capsule sponge testing became a national recommendation, uptake may increase. This is demonstrated by the 10% of the individuals who gave a reason for their non-participation, expressing that they would prefer an endoscopy if they had symptoms that needed investigating.

In the UK lung cancer screening pilot,^24^ 40% of individuals aged 50–75 years identified by their primary care trusts returned a mailed questionnaire designed to assess their eligibility, compared with 48.5% in our study, though a higher proportion were willing to participate (~75% vs 60% in our study), leading both studies to have around 30% of invited individuals who were willing to participate. The range of 22–35% expressing interest in the study among those invited demonstrates the wide variability between sites, and that it may be hard to predict uptake among invited individuals at a national level. A larger proportion of invited individuals who replied were eligible compared with people who self-referred (66% vs 49%), suggesting that inviting individuals may be a more efficient way to screen eligible individuals, since ineligible individuals may ignore the invitation; however, we note the continued challenges with identifying eligible individuals through GP electronic health records. Allowing individuals to self-refer enabled a broader spectrum of at-risk individuals to participate, since the study was not restricted to patients registered with the three GP practices involved in the study, nor to individuals who had a prescription for antireflux medication, since it can be purchased without a prescription. A wider age range was also used for self-referrers; although the number of individuals below age 50 was low, 2/35 (5.7%) were diagnosed with BO. It may be possible to increase eligibility among self-referrers by providing more detailed information on inclusion/exclusion criteria in advertisements or providing a QR code on advertisements where more detailed information is provided.

Overall, the proportion of participants diagnosed with BO was similar for participants who were identified through GP records (4.2%) and those who self-referred (3.4%). We note that the absolute number of cases was low, limiting the power to detect a statistically significant difference, and we caution against reading too much into the difference between the numbers needed to screen per individual with BO detected (24 vs 30). Additionally, there was more loss to follow-up among individuals referred for endoscopy who were invited by their GPs compared with those who self-referred (18% vs 4%, respectively). This likely reflects the health-seeking behaviour of those who self-referred and the older ages of individuals invited by their GPs, who may have more comorbidities.

The PPV of capsule sponge testing in this study was 42%, meaning that 42% of individuals referred for endoscopy based on their positive capsule sponge test were diagnosed with BO. This is lower than the 59% reported in the BEST3 trial,^5^ but higher than the 15%^25^ and 23%^26^ reported in the pilot studies performed by NHS Scotland and NHS England, respectively. The range in PPVs likely reflects differences in the populations in each study, since PPV is dependent on the population prevalence of the condition.^27^ The BEST3 trial was enriched for high-risk individuals, whereas the pilot studies performed by the NHS in Scotland and England included many lower risk individuals. Since individuals who tested negative on the capsule sponge were not offered endoscopy, we were not able to assess the false negative rate in this study, though this has been studied elsewhere.^13 18^ However, this means that we are not able to determine whether the false negative rate differed between individuals who self-referred and those invited by their GPs. Future screening studies that allow recruitment via self-referral could invite a small proportion of individuals who were capsule sponge negative for endoscopy to ensure the rate of false negatives was not substantially higher than among invited individuals.

This study also provided vital information on the practicalities of using mobile units to perform capsule sponge screening. One advantage of using mobile units is that capsule sponges can be offered in multiple locations by the same staff, without needing to train additional people. Additionally, it limited the number of supplies that needed to be available during the study, since all the equipment (eg, capsule sponge devices) was only needed in one location, and there was space to store them in the mobile unit. The mobile unit also provided advertising for the capsule sponge device and publicised the risk factors for OAC, increasing public awareness and allowing individuals to self-refer. Additionally, it did not bring more patients to a GP practice, which was especially important during the COVID-19 pandemic. Since the capsule sponge tests were performed as part of a study rather than routine clinical care, it also meant additional insurance was not required for participating staff at each GP practice. However, the mobile unit was not as comfortable as an indoor facility like a general practice, since it was hot in the summer and cold in the winter; this also made it challenging to store the capsule sponge devices in quality-controlled conditions. The mobile unit did not have toilet facilities, piped water or waste facilities. Additionally, the staff were limited in what other activities they could undertake when there were no patients, unlike in a routine clinic, and there was no backup staff available in case of an adverse event, as there would be in a general practice. There are also costs associated with transporting the mobile unit between locations, which in this study were covered by Heartburn Cancer UK. Since the completion of the study, refinements have been made to the mobile unit to improve ventilation and insulation and to provide air conditioning and an additional method of heating the mobile unit.

### Strengths and limitations

This study has several strengths. The pragmatic nature allowed us to estimate uptake from inviting individuals to participate in a BO screening study, as well as estimate the capacity needed for eligibility evaluation and endoscopies required for follow-up care. It was the first study to allow individuals to self-refer for a capsule sponge test, which proved to be an important route to diagnosing BO. Additionally, we demonstrated that capsule sponge tests can be performed in a mobile unit. A centralised laboratory service ensured quality control of the capsule sponge samples, since the processing and pathology of these samples are not standard for NHS histopathology laboratories.

However, there were also limitations. We only know why a small proportion of individuals contacted by their GPs declined to participate, and we did not have individual-level GP data for individuals who did not respond to the invitation, so it was not possible to compare the characteristics of individuals who did and did not respond. There was some loss to follow-up among individuals referred for endoscopy. We do not know how many people who were eligible to self-refer lived in each area or were exposed to advertising for the study, so it is not possible to assess the proportion of eligible people who self-referred. We were also unable to assess contamination, since individuals invited by their GPs may have also seen advertisements for the study and self-referred rather than responding to their invitation. Additionally, the study was performed at three locations in England; while the self-referral results were similar across the three sites used in the study, this was not the case for the GP-invited individuals, and the results may not be generalisable to other locations or healthcare systems. A cost-effectiveness study was beyond the scope of this study, though previous studies have shown capsule sponge screening to be cost-effective among individuals with GORD.^28 29^

### Comparison to the literature

Almost 95% of people who attempted to swallow a capsule sponge succeeded, similar to other studies.^5 15^ As in earlier studies, capsule sponges were found to be highly acceptable.^5 15–18^ A lower proportion of individuals had a low-confidence negative or equivocal result compared with the BEST3 trial (10.7% vs 19%),^5^ likely reflecting that lab tests were conducted in a research-orientated tissue bank facility in BEST3, compared with a diagnostic ISO-accredited lab with strict quality control in DELTA. Among individuals who were asked to repeat the procedure due to an inadequate sample, 60% participated, a proportion similar to the 65% reported in a previous study.^5^

### Implications for research and practice

The BEST4 Screening Trial, a randomised controlled trial, will evaluate whether capsule sponge screening reduces OAC incidence and mortality in people with reflux who regularly use acid-suppressant medication, compared with usual care (GP referral for endoscopy based on symptoms). Eligibility criteria for BEST4 have been informed by this study.

It would be beneficial to increase the proportion of interested people who are eligible by further refining the search criteria applied to GP electronic health records. Knowing the reasons for ineligibility among individuals identified by GP electronic health records will help further refine search criteria; for example, one-third of interested GP-invited individuals were ineligible due to an upper gastrointestinal endoscopy in the past 5 years; identifying a wider range of search terms to identify these individuals would be beneficial. Self-referral will also be implemented in BEST4, based on results from this study. Among ineligible individuals who self-referred, 43% had not been taking acid suppressants for at least 6 months due to GORD; advertising could be more explicit about the eligibility criteria.

Given the successful use of mobile units in DELTA, lung CT screening and the NHS-Galleri multi-cancer early detection trial, they will be used in BEST4. Lessons from DELTA include that they must be in places where the staff running the unit have access to toilet and water facilities and should be located in areas with high footfall to increase visibility.

## Conclusions

We have demonstrated the feasibility of identifying individuals at increased risk of BO from both GP electronic health records and self-referrals, and that the capsule sponge procedure can be conducted in a mobile unit. We learnt that improving the search criteria of GP electronic health records could improve efficiency, and that while individuals were interested in self-referring, the proportion who were eligible was lower than among interested individuals identified via GP electronic health records. The BO rates were similar among participants who self-referred and those identified through their GP electronic health records, suggesting both routes are useful for case finding.

## Supplementary material

10.1136/bmjgast-2026-002496 online supplemental figure 1

10.1136/bmjgast-2026-002496 online supplemental file 1

10.1136/bmjgast-2026-002496 online supplemental file 2

## Data Availability

Data are available upon reasonable request.
