# Supplementary material for: Using a mobile unit to deliver capsule sponge testing for Barrett’s oesophagus in the community in England through GP invitation and self-referral: an implementation study
Source: BMJ Open Gastroenterol. 2026 Sep 22;13(1):e002496. doi: 10.1136/bmjgast-2026-002496 (PMC13599947; doi:10.1136/bmjgast-2026-002496)
Supplement: Supplementary file 1 [file bmjgast-13-1-s001.pdf]

HEARTBURN CANCER UK

HEART01F A

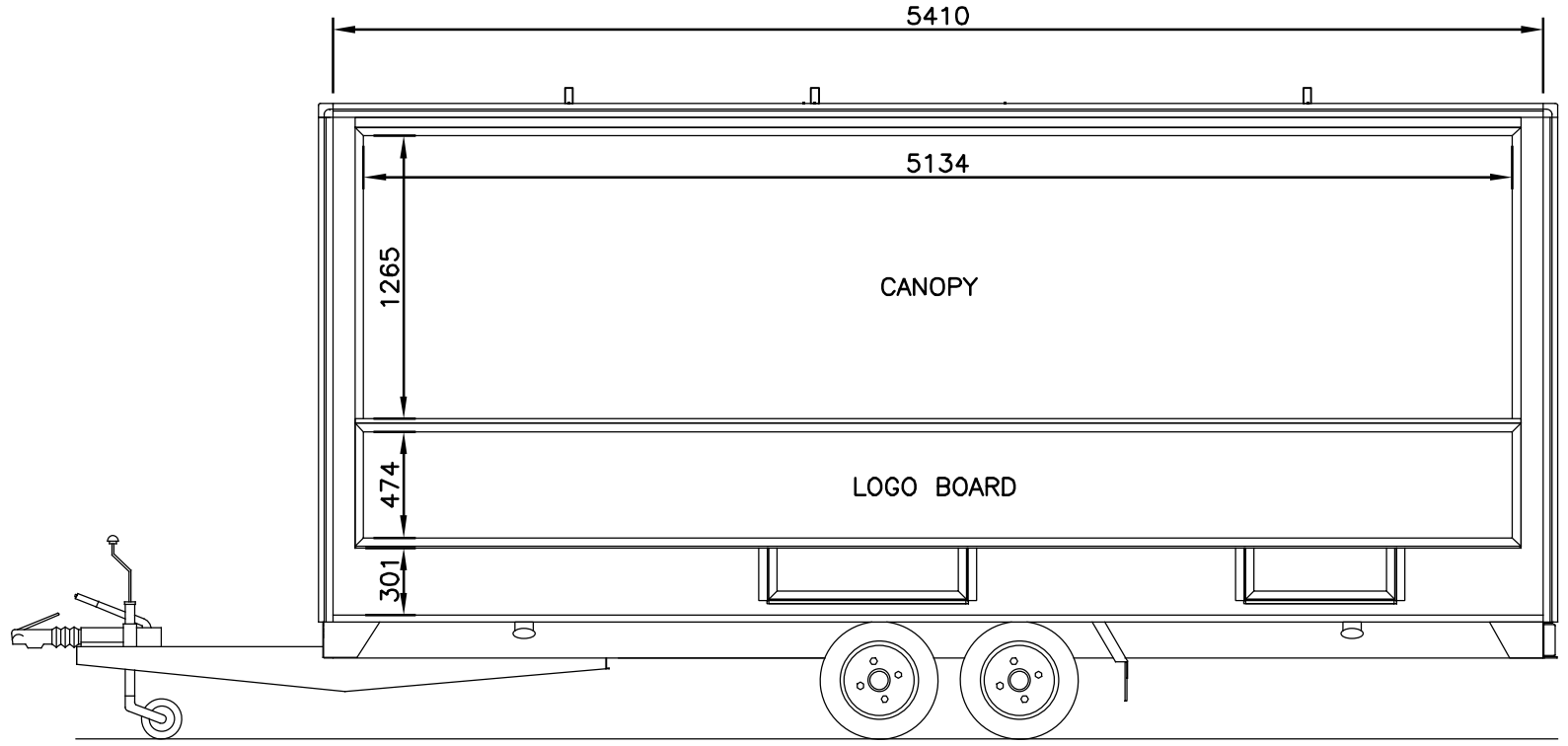

NEARSIDE VIEW (CLOSED)

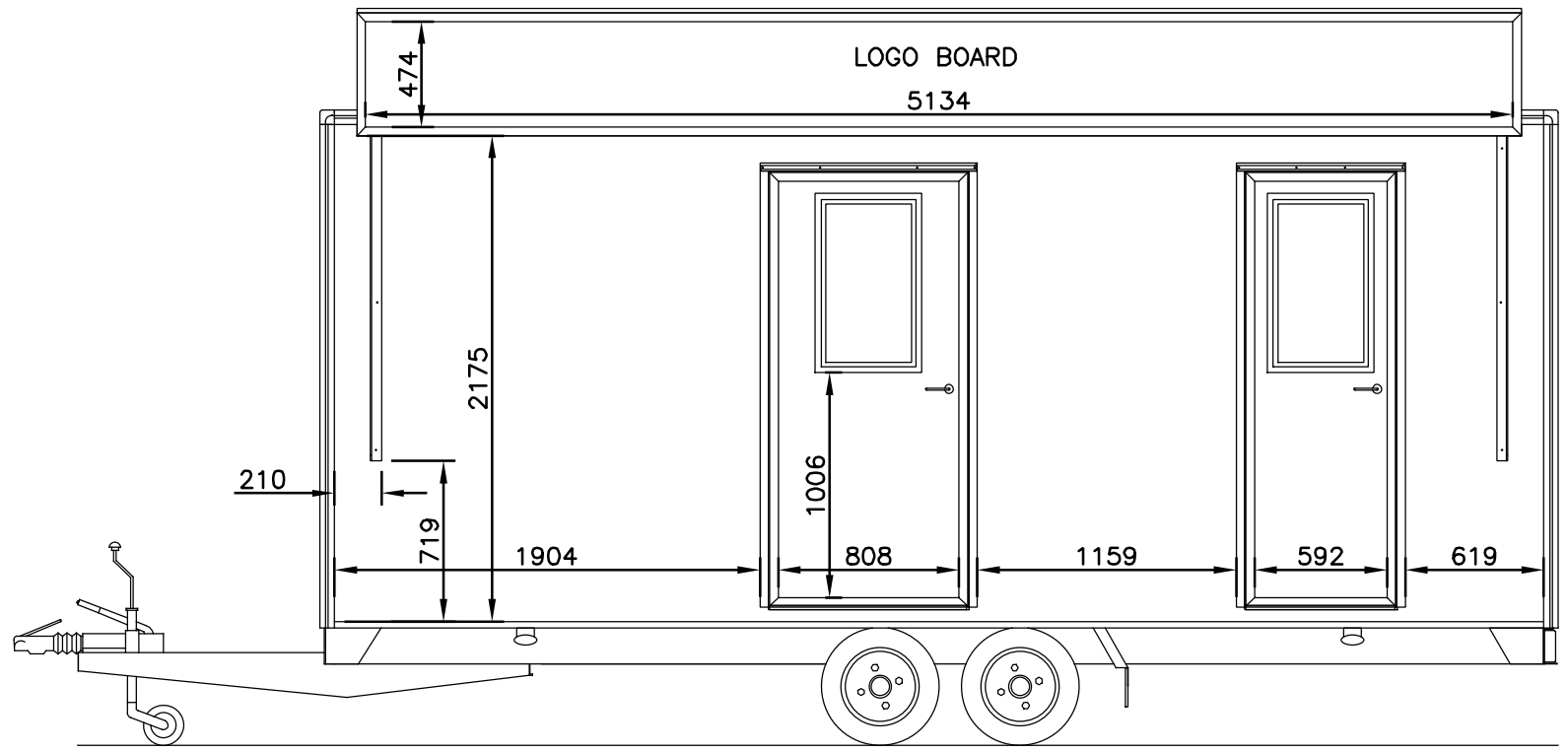

NEARSIDE VIEW (OPEN)

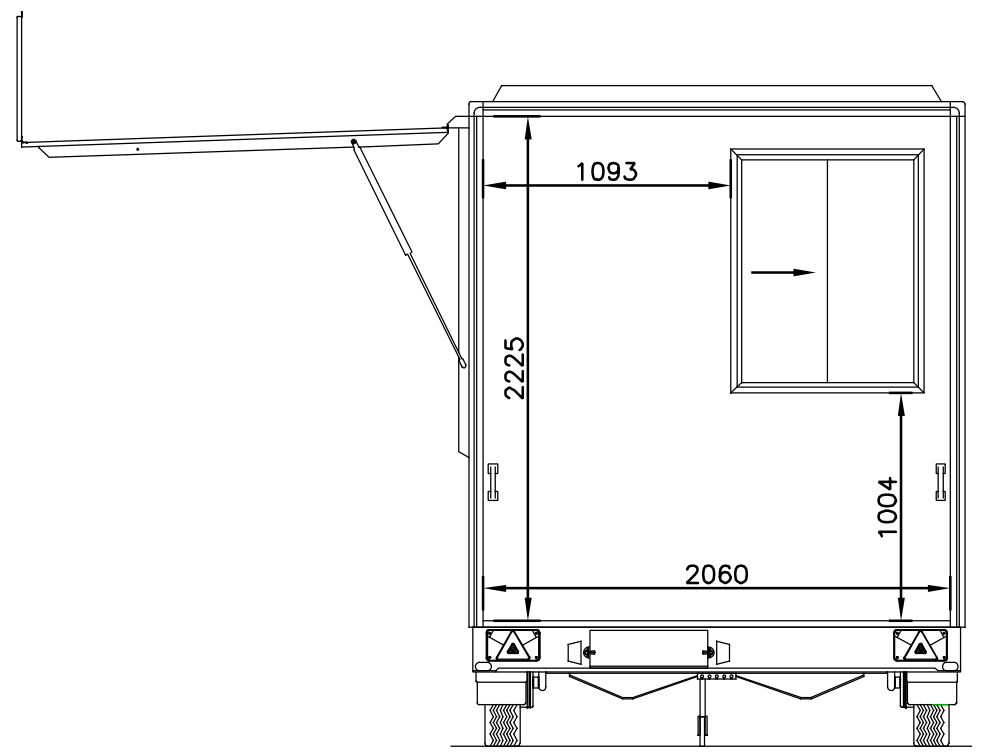

REAR VIEW (OPEN)

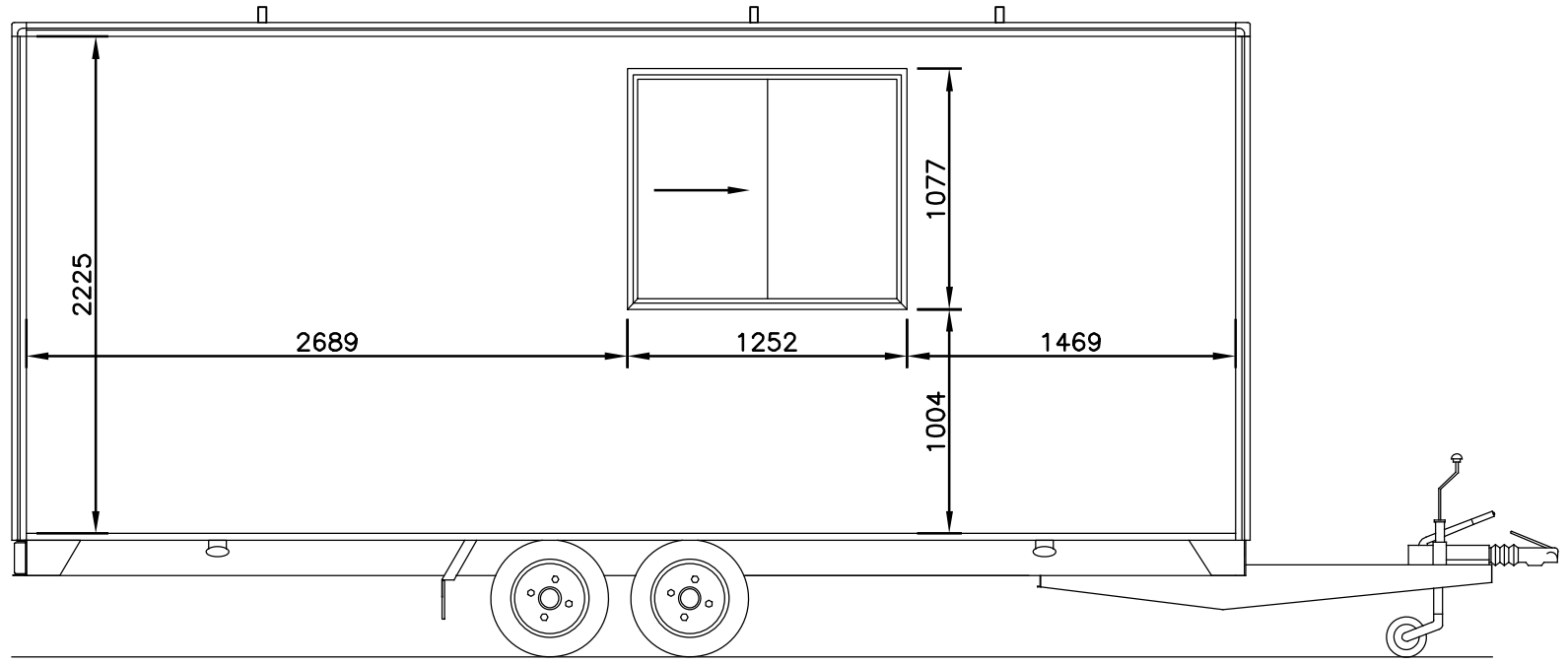

OFFSIDE VIEW

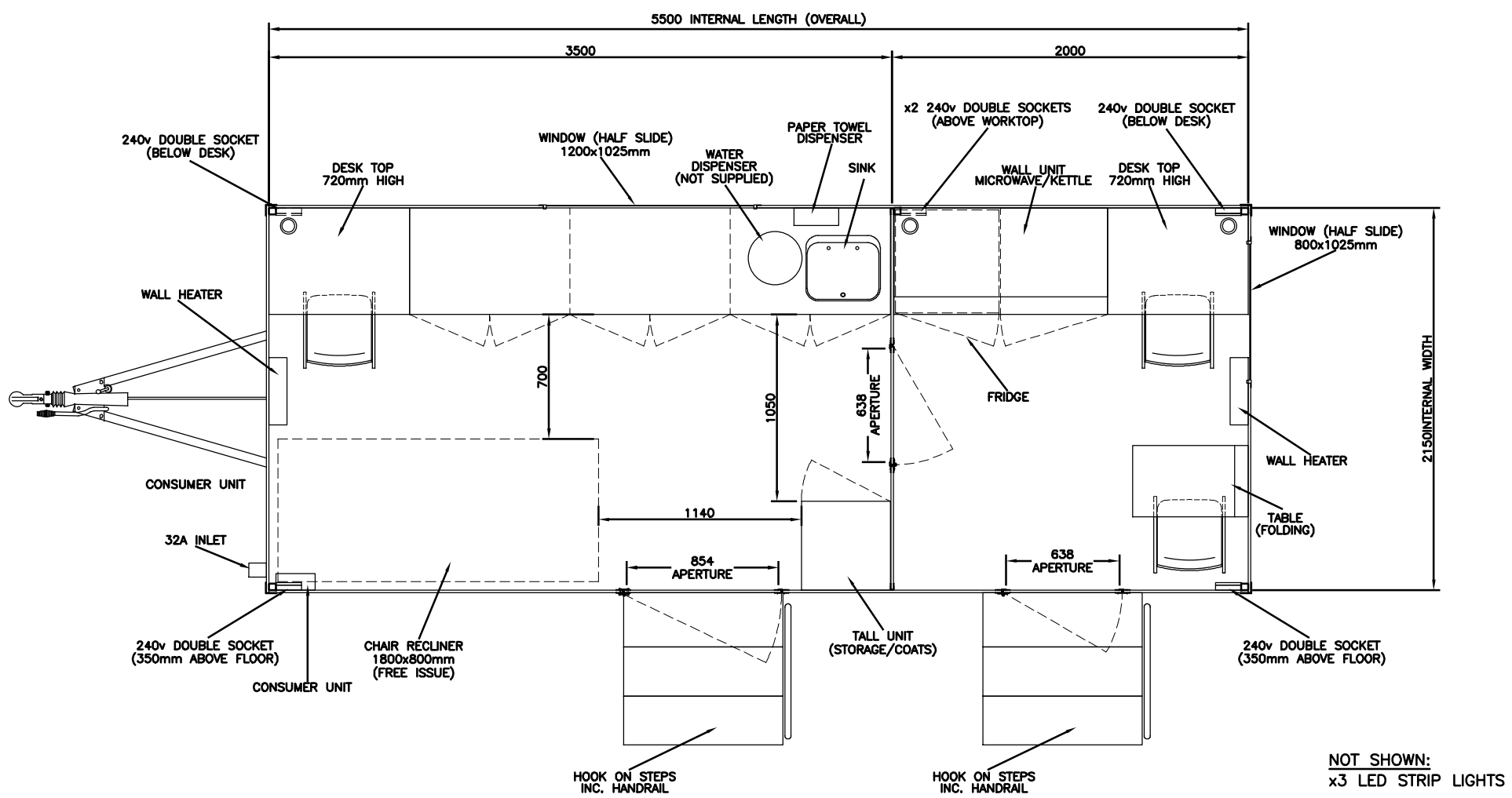

PLAN VIEW

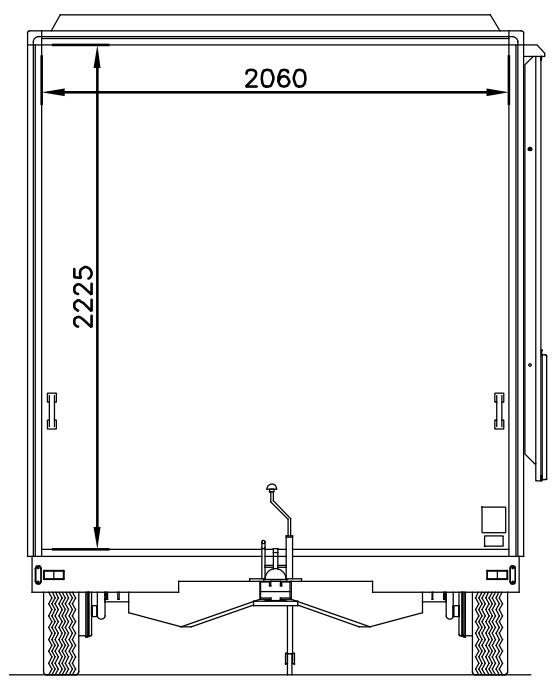

FRONT VIEW (CLOSED)

TOW MASTER

5500 x 2150MM MOBILE DIAGNOSTIC UNIT

LAYOUT & DECAL
