# Supplementary material for: Using a mobile unit to deliver capsule sponge testing for Barrett’s oesophagus in the community in England through GP invitation and self-referral: an implementation study
Source: BMJ Open Gastroenterol. 2026 Sep 22;13(1):e002496. doi: 10.1136/bmjgast-2026-002496 (PMC13599947; doi:10.1136/bmjgast-2026-002496)
Supplement: Supplementary file 2 [file bmjgast-13-1-s002.docx]

**Supplementary Methods**

*Study sites and mobile unit settings*

Since the capsule sponge procedure had not previously been conducted in a mobile unit, this study was piloted in a general practice car park in Shelford, where medical support was available and a hospital close-by in case of emergency. The second site, in Chelmsford, was a Sports Centre outside the town centre, around 100m from the general practice, and in an area with a diverse population; this site was much more noticeable to the general public, had free parking, and was sometimes used as the site of the mobile breast screening unit. It was also close to the running track and the park, and dog walkers and passers-by using the sports centre facilities enquired about the study. Halfway through the time in Chelmsford, the unit had to move nearer to the running track, to a more secluded spot which lacked visibility to the public, due to the mobile breast screening unit returning earlier than anticipated. The third site in Eye was in the grounds of a community hospital on a hill near a main road, where the mobile breast screening unit used to be, and where passers-by in their cars could see the unit. Additionally, the unit had access to community hospital facilities for water supply and toilets facilities. This site was 50m from one of the general practices in the study and was not far from the town centre.

*Study procedures on the mobile unit*

The mobile unit was staffed by two individuals, of whom at least one was a nurse, with the other being a nurse, project manager or administrator. Although the mobile unit was not wheelchair accessible, if a patient required this, they were seen in a clinic room nearby, for example in the first aid room at the sports centre in Chelmsford. Up to 15 appointments could be scheduled per day, though 8-12 was standard. Overall, individuals did not show up for <1% of the scheduled appointments due to telephone reminders. Over 80% of appointments lasted between 16-30 minutes, with 9% lasting <15 minutes. Before leaving the mobile unit, participants were given some after care information and a Heartburn Cancer UK booklet containing information about their symptoms.

*Acceptability questions*

Participants completed a questionnaire after the procedure, including two questions on acceptability: “How acceptable did you find the Cytosponge procedure?” (completely acceptable, acceptable, unacceptable, completely unacceptable), and “Would you be willing to have the Cytosponge procedure again in the future, if it was recommended by your doctor?” (yes, definitely; yes, probably; unsure; no, probably not; no, definitely not).

**Supplementary Results**

*Acceptability*

Of the 749 individuals who attempted to swallow a capsule sponge, 712 responded to the question “how acceptable did you find the Cytosponge procedure?”: 407 (57.2%) said the procedure was completely acceptable, 301 (42.3%) saif it was acceptable, 1 (0.1%) said it was unacceptable, and 3 (0.4%) said it was completely unacceptable.

Of the 749 individuals who attempted to swallow a capsule sponge, 714 responded to the question “Would you be willing to have the Cytosponge procedure again in the future, if it was recommended by your doctor?”: 479 (67.1%) responded ‘yes, definitely’, 212 (29.7%) responded ‘yes, probably’, 12 (1.7%) responded ‘unsure’, 7 (1.0%) responded ‘no, probably not’, and 4 (0.6%) responded ‘no, definitely not’.

*Repeat capsule sponge uptake by acceptability*

Of the 76 individuals invited for a repeat capsule sponge, 59 (77.6%) had reported “yes, definitely” to whether they would be willing to do the capsule sponge again at their original appointment, 16 (21.1%) had responded “yes, probably” and 1 (1.3%) had said they were unsure. Yet 25/76 (32.9%) declined a repeat capsule sponge following an inadequate result. Overall, 45 of the 76 individuals (59.2%) attended an appointment for a repeat capsule sponge: of the 58 who said ‘yes, definitely’, 37 (63.8%) attended an appointment for a repeat capsule sponge, of whom 35 successfully swallowed a capsule sponge, 19 (32.7%) declined a repeat capsule sponge, 2 did not reply, and 1 attended endoscopy. Of the 16 who said ‘yes, probably’, 8 (50.0%) attended and successfully swallowed a capsule sponge, 5 (31.2%) declined and 3 did not reply.

**Supplementary Table 1**: Study participation by study site and invitation status (invited by GP vs self-referral)

| **GP** | Shelford | Suffolk | Chelmsford | Total |
| --- | --- | --- | --- | --- |
| Invited by GP | 850 | 1203 | 1240 | 3293 |
| Responded to invite NO | 151 | 241 | 254 | 646 |
| Responded to invite YES | 295 | 388 | 269 | 952 |
| % who responded | 52% | 52% | 42% | 49% |
| of those who responded, % who said yes | 66% | 62% | 51% | 60% |
|  |  |  |  |  |
| Ineligible to participate | 62 | 131 | 99 | 292 |
| Eligible to participate | 221 | 213 | 140 | 574 |
| Could not be contacted/contacted too late to assess eligibility, or changed their mind | 12 | 44 | 30 | 86 |
| % eligible among those who responded | **78%** | **62%** | **59%** | **66%** |
|  |  |  |  |  |
| Withdrew before procedure | 35 | 7 | 0 | 42 |
| agreed to participate | 186 | 206 | 140 | 532 |
| % of those eligible who agreed to participate | **84%** | **97%** | **100%** | **93%** |
| Successfully swallowed a capsule sponge | 178 | 193 | 131 | 502 |
| Unable to swallow | 8 | 13 | 9 | 30 |
| % of those who agreed to participate who swallowed capsule sponge | 96% | 94% | 94% | 94% |
| % of those invited who swallowed capsule sponge | **21%** | **16%** | **11%** | **15%** |
|  |  |  |  |  |
| **Self-referrals** | Shelford | Suffolk | Chelmsford | Total |
| Number who contacted for more information | 191 | 86 | 248 | 525 |
| Number eligible to participate | 86 | 36 | 105 | 227 |
| Could not be contacted/contacted too late to assess eligibility, or changed their mind | 17 | 10 | 30 | 57 |
| % eligible of those who contacted study | **45%** | **42%** | **42%** | **43%** |
|  |  |  |  |  |
| Agreed to participate | 79 | 35 | 103 | 217 |
| Successfully swallowed a capsule sponge | 77 | 33 | 98 | 208 |
| Unable to swallow | 2 | 2 | 5 | 9 |
| % of those who agreed to participate who swallowed capsule sponge | 97% | 94% | 95% | 96% |
| % of those who contacted study who swallowed capsule sponge | **40%** | **38%** | **40%** | **40%** |

**Supplementary Table 2**: Reasons for ineligibility, by invitation status and study site.

|  | Total | | GP-invited | | | | | | | | Self-referrals | | | | | | | |
| --- | --- | --- | --- | --- | --- | --- | --- | --- | --- | --- | --- | --- | --- | --- | --- | --- | --- | --- |
|  |  |  | Chelmsford | | Shelford | | Suffolk | | Total | | Chelmsford | | Shelford | | Suffolk | | Total | |
|  | N | % | N | % | N | % | N | % | N | % | N | % | N | % | N | % | N | % |
| Upper GI issue | 21 | 4.8% | 4 | 4.2% | 3 | 5.2% | 6 | 4.8% | 13 | 4.7% | 7 | 6.4% | 0 | 0.0% | 1 | 3.1% | 8 | 5.1% |
| On contra-indicated medications | 48 | 11.0% | 18 | 18.9% | 2 | 3.4% | 20 | 15.9% | 40 | 14.3% | 5 | 4.6% | 2 | 12.5% | 1 | 3.1% | 8 | 5.1% |
| PPI was co-prescribed | 53 | 12.2% | 17 | 17.9% | 17 | 29.3% | 17 | 13.5% | 51 | 18.3% | 2 | 1.8% | 0 | 0.0% | 0 | 0.0% | 2 | 1.3% |
| Comorbidity | 66 | 15.1% | 15 | 15.8% | 12 | 20.7% | 20 | 15.9% | 47 | 16.8% | 10 | 9.2% | 5 | 31.3% | 4 | 12.5% | 19 | 12.1% |
| Recent endoscopy | 148 | 33.9% | 36 | 37.9% | 7 | 12.1% | 52 | 41.3% | 95 | 34.1% | 37 | 33.9% | 6 | 37.5% | 10 | 31.3% | 53 | 33.8% |
| Not on PPI/insufficient reflux symptoms | 100 | 22.9% | 5 | 5.3% | 17 | 29.3% | 11 | 8.7% | 33 | 11.8% | 48 | 44.0% | 3 | 18.8% | 16 | 50.0% | 67 | 42.7% |
| Total | 436 | 100% | 95 | 100% | 58 | 100% | 126 | 100% | 279 | 100% | 109 | 100% | 16 | 100% | 32 | 100% | 157 | 100% |

**Supplementary Figure 1**: A schematic of the mobile unit

See separate supplement. Schematic provided by Heartburn Cancer UK.

**Supplementary Figure 2**: The outside of the mobile unit

Photo provided by Heartburn Cancer UK
